# Supplementary material for: Generation and characterization of cardiac valve endothelial-like cells from human pluripotent stem cells
Source: Commun Biol. 2021 Sep 6;4:1039. doi: 10.1038/s42003-021-02571-7 (PMC8421482; doi:10.1038/s42003-021-02571-7)
Supplement: Supplementary file 2 — Supplementary Information [file 42003_2021_2571_MOESM2_ESM.pdf]

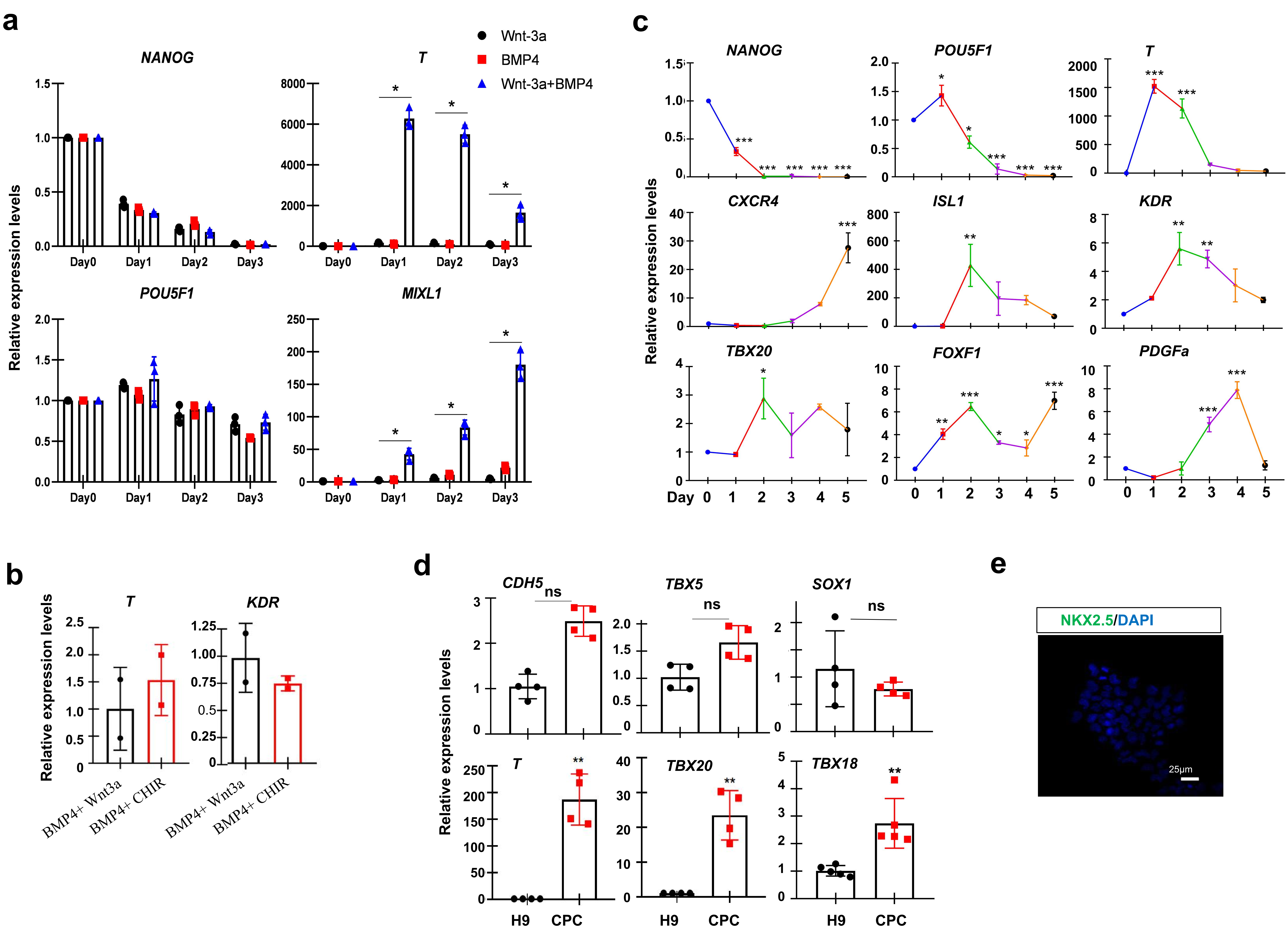
a
Wnt-3a
BMP4
Wnt-3a+BMP4
NANOG
T
POU5F1
MIXL1
Relative expression levels
Day0
Day1
Day2
Day3
b
T
KDR
Relative expression levels
BMP4+ Wnt3a
BMP4+ CHIR
c
NANOG
POU5F1
T
CXCR4
ISL1
KDR
TBX20
FOXF1
PDGFa
Relative expression levels
Day
d
CDH5
TBX5
SOX1
T
TBX20
TBX18
ns
Relative expression levels
H9
CPC
e
NKX2.5/DAPI
25μm

Supplementary Figure 1. Related to Figure 1. **a** The qRT-PCR analysis of indicated genes for hPSCs treated with BMP4 and Wnt3a for a time course of 3 days. **b** The qRT-PCR analysis of indicated genes, showing that Wnt3a/BMP4 treatment had a similar effects on gene expression to CHIR/BMP4 treatment. **c** Day 1 hPSC-derived *T* positive cells were treated with bFGF and BMP4 for a time course of 5 days, and the expression of indicated genes were examined daily. **d** The qRT-PCR analysis of day 3 hPSC-derived CPCs for the indicated markers. **e** IF staining of day 3 hPSC-derived CPCs for NKX2.5. All experiments were repeated 3 times. The paired t test in Graphpad software was used for the statistical analysis. Significant levels are: *$p< 0.05$; **$P< 0.01$; ***$P< 0.001$. Shown are representative data for panel e.

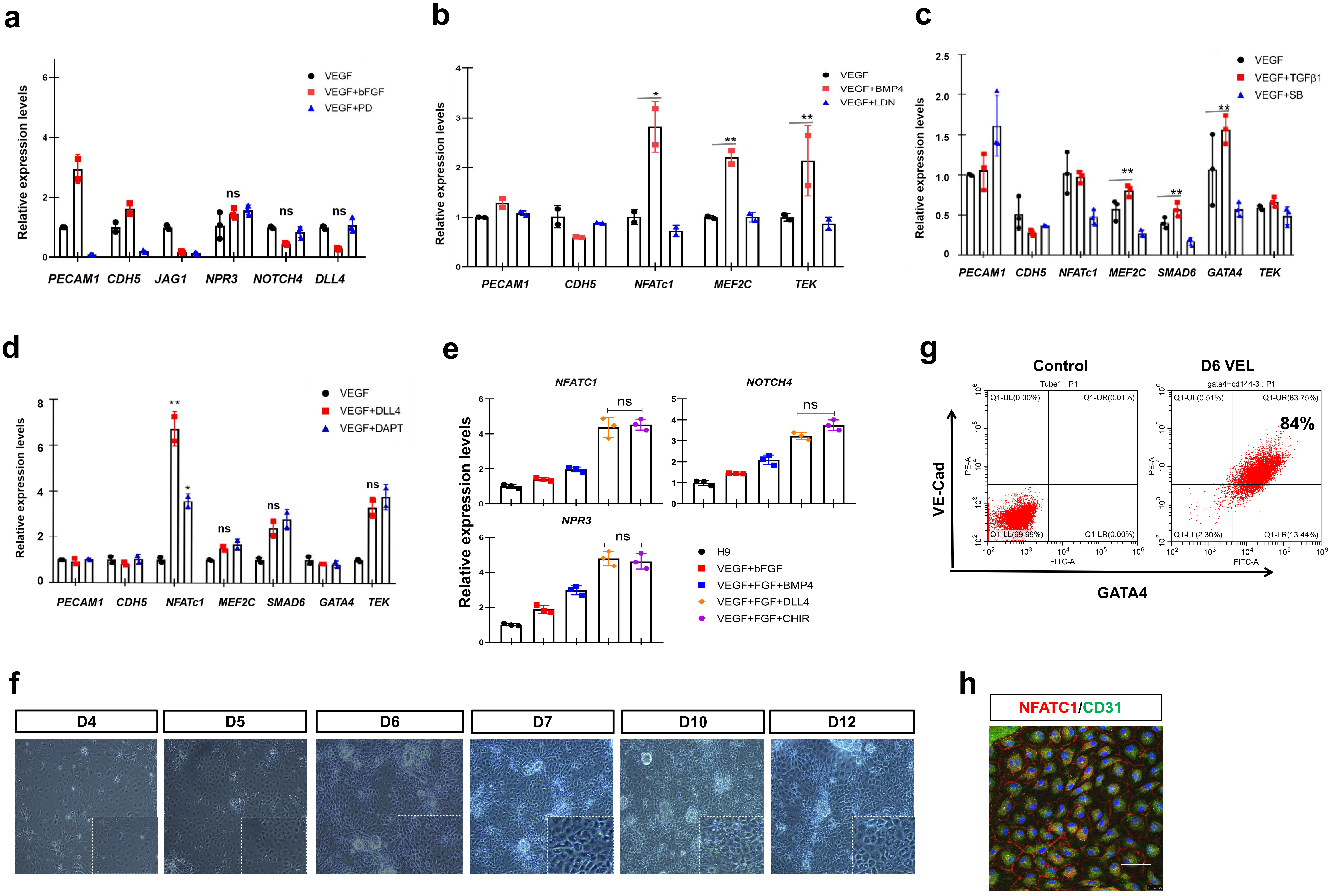
a
Relative expression levels
VEGF
VEGF+bFGF
VEGF+PD
ns
PECAM1 CDH5 JAG1 NPR3 NOTCH4 DLL4
b
Relative expression levels
VEGF
VEGF+BMP4
VEGF+LDN
PECAM1 CDH5 NFATc1 MEF2C TEK
c
Relative expression levels
VEGF
VEGF+TGFβ1
VEGF+SB
PECAM1 CDH5 NFATc1 MEF2C SMAD6 GATA4 TEK
d
Relative expression levels
VEGF
VEGF+DLL4
VEGF+DAPT
ns
PECAM1 CDH5 NFATc1 MEF2C SMAD6 GATA4 TEK
e
Relative expression levels
NFATC1
NOTCH4
NPR3
ns
H9
VEGF+bFGF
VEGF+FGF+BMP4
VEGF+FGF+DLL4
VEGF+FGF+CHIR
g
Control
D6 VEL
Tube1 : P1
gata4+cd144-3 : P1
Q1-UL(0.00%)
Q1-UR(0.01%)
Q1-LL(99.99%)
Q1-LR(0.00%)
Q1-UL(0.51%)
Q1-UR(83.75%)
84%
Q1-LL(2.30%)
Q1-LR(13.44%)
PE-A
FITC-A
VE-Cad
GATA4
f
D4
D5
D6
D7
D10
D12
h
NFATC1/CD31

Supplementary Figure 2. Related to Figure 2. **a** The qRT-PCR analysis of indicated genes for CPCs that treated with FGF inhibitor PD or bFGF for 3 days. **b** The qRT-PCR analysis of indicated genes for CPCs that treated with BMP4 or BMP inhibitor LDN for 3 days. **c** The qRT-PCR analysis of indicated genes for CPCs that treated with TGFb1 or TGF inhibitor SB for 3 days. **d** The qRT-PCR analysis of indicated genes for CPCs that treated with NOTCH inhibitor DAPT or activator DLL4 for 3 days. **e** The qRT-PCR analysis of indicated genes, showing that DLL4 addition did not augment the expression of ECC genes, in the presence of VAGFA/BMP4/TGFb1. **f** The representative morphology of hPSC-derived CPCs treated with VEGFA, TGFb1 and BMP4 for a time course of 12 days. **g** Flow cytometry analysis showing the percentage of GATA4/VE-cad double positive cells in day 5 hPSC-derived VELs. **h** IF staining of day 8 hPSC-derived VELs showing the co-expression of VEC-specific marker NFATc1 and CD31. Scale bar: 25 μm. The paired t test in Graphpad software was used for the statistical analysis. Significant levels are: $*p < 0.05$; $**P < 0.01$; $***P < 0.001$. Shown are representative images for panels f and h.

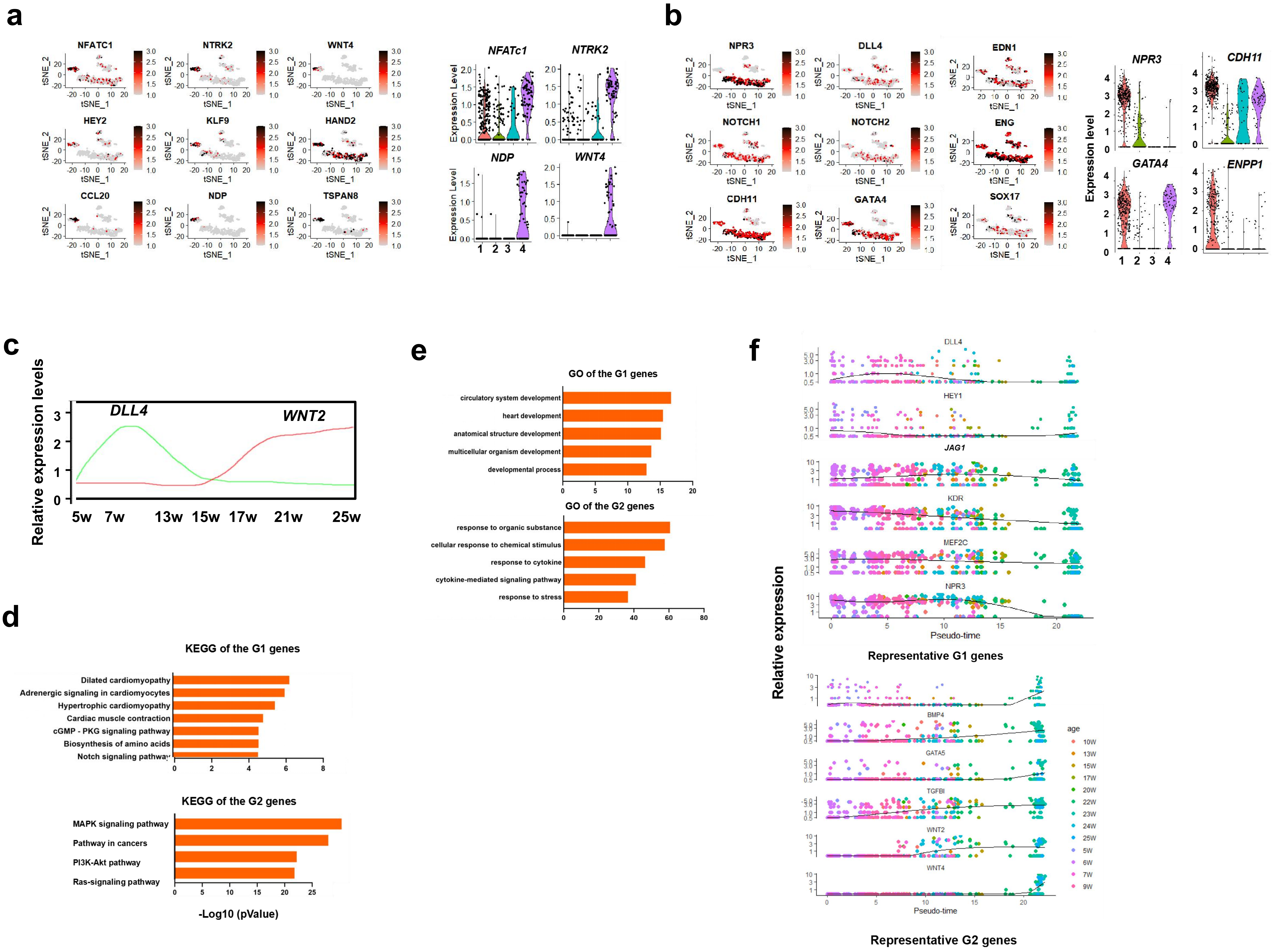
a
NFATC1
NTRK2
WNT4
HEY2
KLF9
HAND2
CCL20
NDP
TSPAN8
tSNE_1
tSNE_2
NFATc1
NTRK2
NDP
WNT4
Expression Level
1 2 3 4
b
NPR3
DLL4
EDN1
NOTCH1
NOTCH2
ENG
CDH11
GATA4
SOX17
NPR3
CDH11
GATA4
ENPP1
Expression level
1 2 3 4
c
Relative expression levels
DLL4
WNT2
5w 7w 13w 15w 17w 21w 25w
d
KEGG of the G1 genes
Dilated cardiomyopathy
Adrenergic signaling in cardiomyocytes
Hypertrophic cardiomyopathy
Cardiac muscle contraction
cGMP - PKG signaling pathway
Biosynthesis of amino acids
Notch signaling pathway
KEGG of the G2 genes
MAPK signaling pathway
Pathway in cancers
PI3K-Akt pathway
Ras-signaling pathway
-Log10 (pValue)
e
GO of the G1 genes
circulatory system development
heart development
anatomical structure development
multicellular organism development
developmental process
GO of the G2 genes
response to organic substance
cellular response to chemical stimulus
response to cytokine
cytokine-mediated signaling pathway
response to stress
f
DLL4
HEY1
JAG1
KDR
MEF2C
NPR3
Pseudo-time
Representative G1 genes
Relative expression
BMP4
GATA5
TGFBI
WNT2
WNT4
Pseudo-time
age
10W
13W
15W
17W
20W
22W
23W
24W
25W
5W
6W
7W
9W
Representative G2 genes

Supplementary Figure 3. a Left panel: tSNE maps showing the expression of indicated VEC marker genes in the identified VEC cluster; right panel: violin plots showing expression of indicated genes in all EC clusters (see in Figure 3a). **b** Left: tSNE maps showing expression of the indicated ECC marker genes in the identified endocardium; right panel: violin plots showing expression of the indicated genes in all EC clusters. **c** Cartoon showing the dynamic expression of the representative G1 (*Dll4*) and G2 (*WNT2*) genes, based on developmental timing. **d** KEGG analysis of G1 and G2 genes. **e** GO analysis of G1 and G2 genes. **f** Dynamic expression of representative G1 and G2 genes in their respective branches of endocardial cells and VECs at different developmental stages. Note that the representative G1 genes are expressed in endocardium of early developmental stage, and the representative G2 genes are expressed in VECs of late stage.

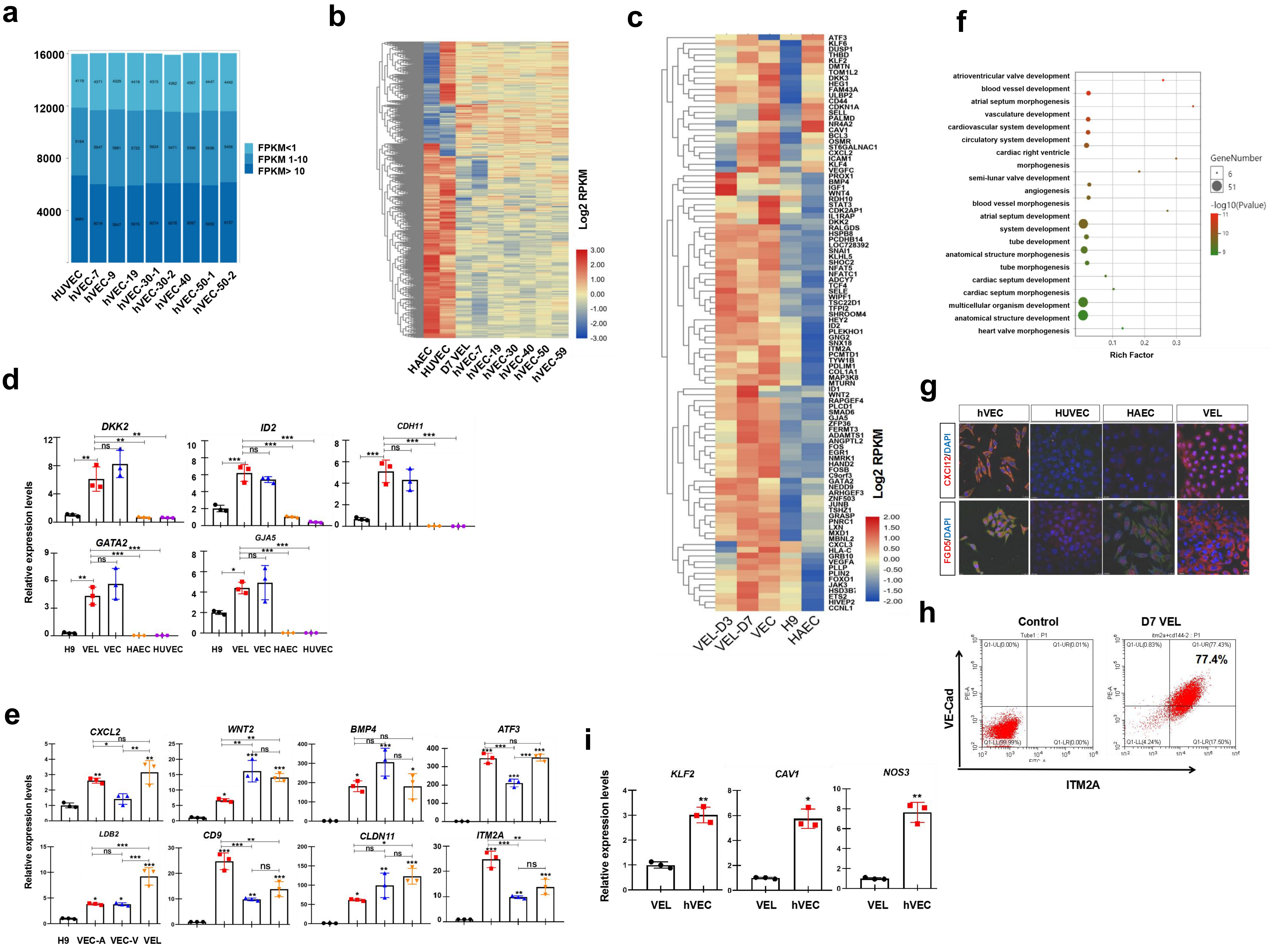
a
16000
12000
8000
4000
FPKM<1
FPKM 1-10
FPKM> 10
HUVEC
hVEC-7
hVEC-9
hVEC-19
hVEC-30-1
hVEC-30-2
hVEC-40
hVEC-50-1
hVEC-50-2
b
Log2 RPKM
3.00
2.00
1.00
0.00
-1.00
-2.00
-3.00
HAEC
HUVEC
D7 VEL
hVEC-7
hVEC-19
hVEC-30
hVEC-40
hVEC-50
hVEC-59
c
ATF3 KLF6 DUSP1 THBD KLF2 DMTN TOM1L2 DKK3 HEG1 FAM43A ULBP2 CD44 CDKN1A SELL PALMD NR4A2 CAV1 BCL3 OSMR ST6GALNAC1 CXCL2 ICAM1 KLF4 VEGFC PROX1 BMP4 IGF1 WNT4 RDH10 STAT3 CDK2AP1 IL1RAP DKK2 RALGDS HSPB8 PCDHB14 LOC728392 SNAI1 KLHL5 SHOC2 NFAT5 NFATC1 ADCY7 TCF4 SELE WIPF1 TSC22D1 TFPI2 SHROOM4 HEY2 ID2 PLEKHO1 GNG2 SNX18 ITM2A PCMTD1 TYW1B PDLIM1 COL1A1 MAP3K8 MTURN ID1 WNT2 RAPGEF4 PLCD1 SMAD6 GJA5 ZFP36 FERMT3 ADAMTS1 ANGPTL2 FOS EGR1 NMRK1 HAND2 FOSB C9orf3 GATA2 NEDD9 ARHGEF3 ZNF503 JUNB TSHZ1 GRASP PNRC1 LXN MXD1 MBNL2 CXCL3 HLA-C GRB10 VEGFA PLLP PLIN2 FOXO1 JAK3 HSD3B7 ETS2 HIVEP2 CCNL1
Log2 RPKM
2.00
1.50
1.00
0.50
0.00
-0.50
-1.00
-1.50
-2.00
VEL-D3
VEL-D7
VEC
H9
HAEC
f
atrioventricular valve development
blood vessel development
atrial septum morphogenesis
vasculature development
cardiovascular system development
circulatory system development
cardiac right ventricle
morphogenesis
semi-lunar valve development
angiogenesis
blood vessel morphogenesis
atrial septum development
system development
tube development
anatomical structure morphogenesis
tube morphogenesis
cardiac septum development
cardiac septum morphogenesis
multicellular organism development
anatomical structure development
heart valve morphogenesis
0.1
0.2
0.3
Rich Factor
GeneNumber
6
51
-log10(Pvalue)
11
10
9
d
Relative expression levels
DKK2
ID2
CDH11
GATA2
GJA5
H9
VEL
VEC
HAEC
HUVEC
e
Relative expression levels
CXCL2
WNT2
BMP4
ATF3
LDB2
CD9
CLDN11
ITM2A
H9
VEC-A
VEC-V
VEL
g
hVEC
HUVEC
HAEC
VEL
CXCl12/DAPI
FGD5/DAPI
h
Control
D7 VEL
Q1-UL(0.00%)
Q1-UR(0.01%)
Q1-LL(99.99%)
Q1-LR(0.00%)
Q1-UL(0.83%)
Q1-UR(77.43%)
Q1-LL(4.24%)
Q1-LR(17.50%)
77.4%
VE-Cad
ITM2A
i
Relative expression levels
KLF2
CAV1
NOS3
VEL
hVEC

Supplementary Figure 4. Related to Figure 4. **a** Representative image showing the gene numbers and gene expression levels of HUVEC and the primary VECs isolated from aortic valves of different ages. For instance, hVEC-30-1 and hVEC-30-2 are human aortic valve endothelial cells isolated from two 30-year-old individuals. **b** Heat map showing the relationship among HAEC, HUVEC, the primary VECs from different ages and day 7 hPSC-derived VELs. Note that day 7 hPSC-derived VELs were more similar to the primary VECs than HUVEC and HAEC. **c** Heat map of G2 genes in hPSC-derived VELs (day 3 and 7), the primary VECs of 9-year old, H9 and HAEC, showing that G2 genes are expressed higher in VEL and VECs than in HAEC. **d-e** The qRT-PCR analysis of the selected marker genes for (c). hVEC-A and hVEC-V stand for the primary VECs isolated from the aortic side and the ventricular side of valves, respectively. **f** KEGG analysis of shared genes between day 7 hPSC-derived VELs and the primary hVECs of 9-year old. **g** IF staining results showing that FGD5 and CXCL12 are abundantly expressed in the primary VECs and day 7 hPSC-derived VELs, but lowly expressed in HUVEC and HAEC. **h** Flow cytometry analysis of ITM2A/VE-cad double positive cells in day 7 hPSC-derived VELs, indicating that ITM2A might be a useful VEC surface marker. Left: isotype control. **i** The qRT-PCR analysis of selected oscillatory shear-related genes, showing that they were expressed at lower levels in day 7 hPSC-derived VELs than in primary VECs. All experiments were repeated 3 times. The paired t test in Graphpad software was used for the statistical analysis. Significant levels are: $*p < 0.05$; $**P < 0.01$; $***P < 0.001$.

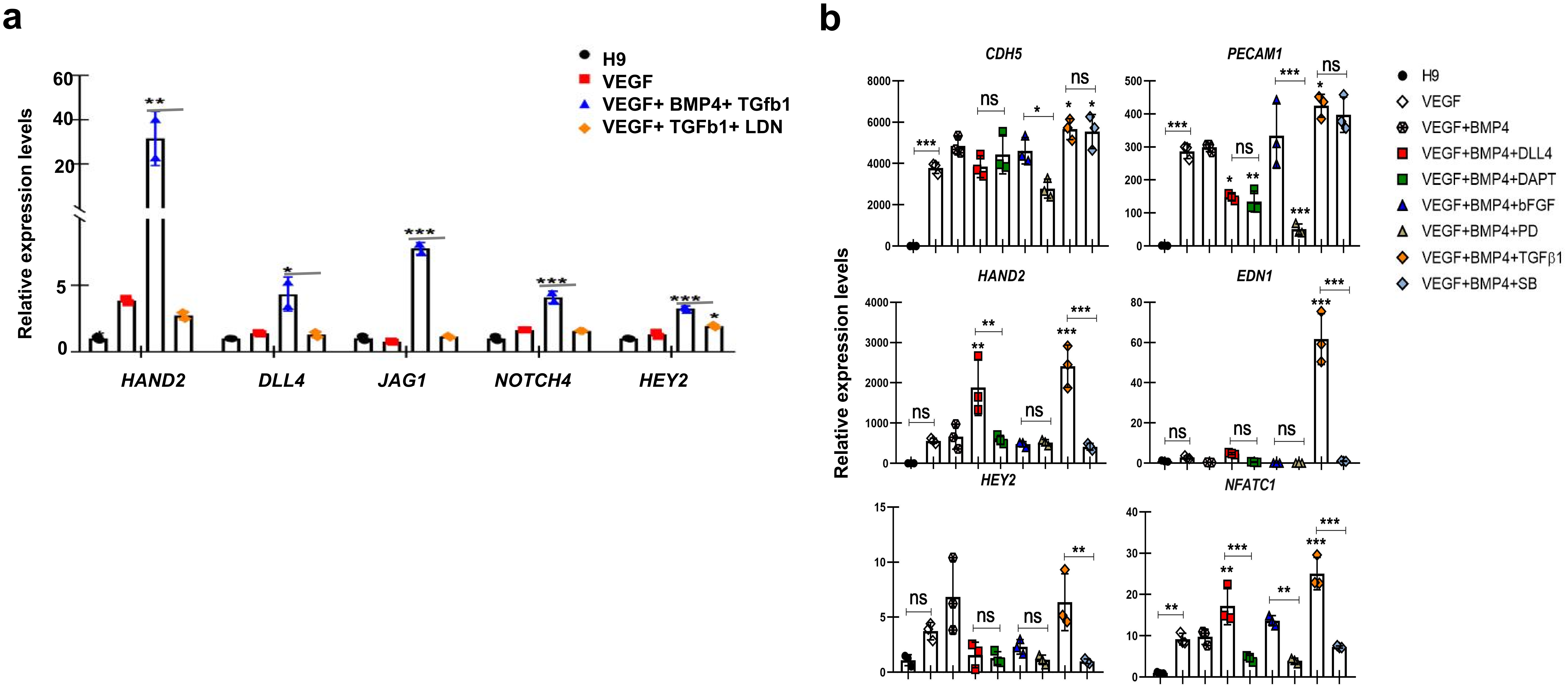

Supplementary Figure 5. Related to Figure 5. **a** The qRT-PCR analysis of selected NOTCH-related genes. Note that expression of NOTCH-related genes was decreased when BMP signaling was inhibited by addition of LDN. **b** The qRT-PCR analysis of indicated genes in various signaling conditions. Note that expression of *NFATc1/HEY2/HAND2/EDN1* was greatly decreased when TGFβ signaling was inhibited by the addition of SB. The paired t test in Graphpad software was used for the statistical analysis. Significant levels are: *$p < 0.05$; **$P < 0.01$; ***$P < 0.001$. ns: not significant. All experiments were repeated 3 times.

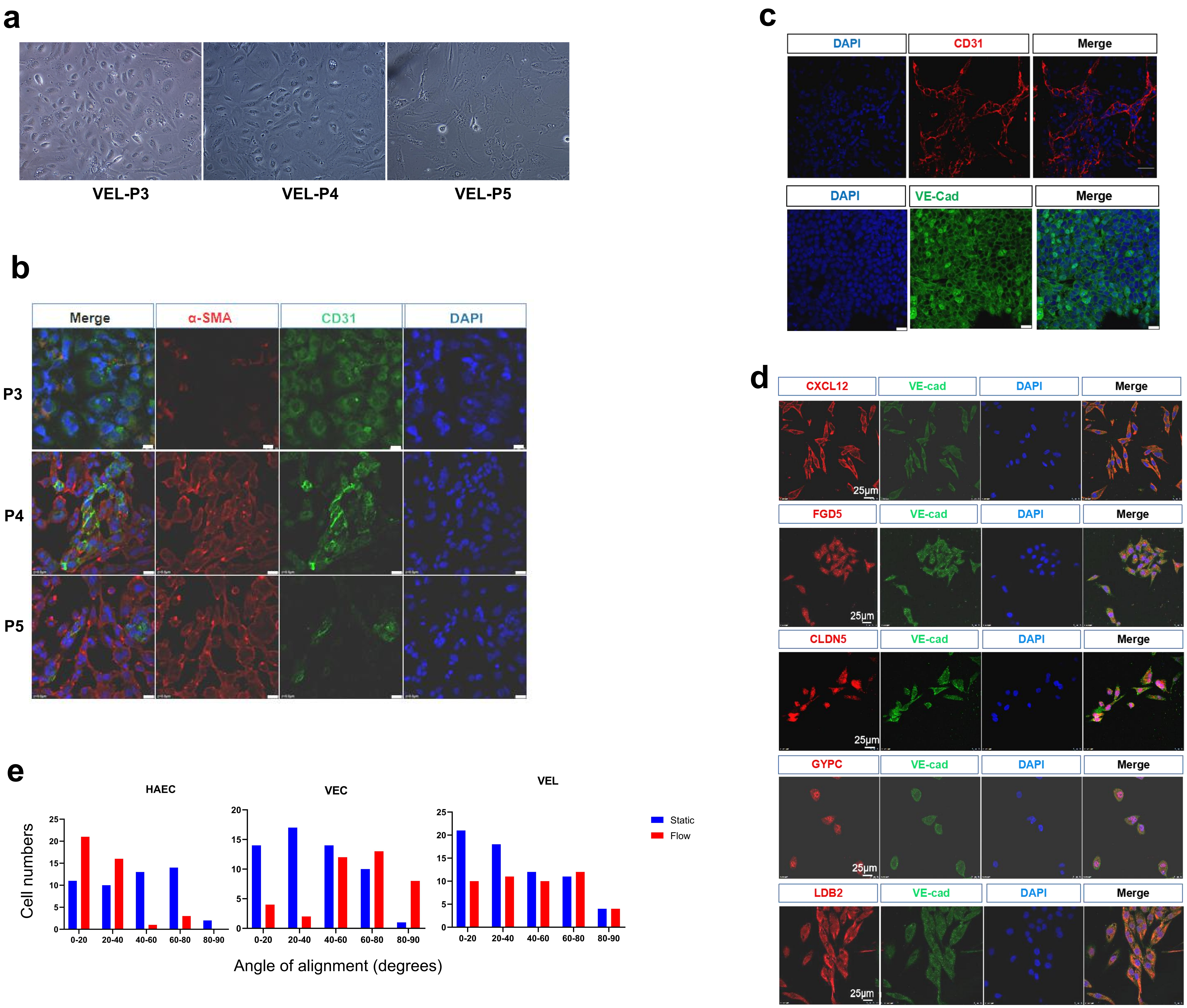
a
VEL-P3
VEL-P4
VEL-P5
b
Merge
α-SMA
CD31
DAPI
P3
P4
P5
c
DAPI
CD31
Merge
DAPI
VE-Cad
Merge
d
CXCL12
VE-cad
DAPI
Merge
25μm
FGD5
VE-cad
DAPI
Merge
25μm
CLDN5
VE-cad
DAPI
Merge
25μm
GYPC
VE-cad
DAPI
Merge
25μm
LDB2
VE-cad
DAPI
Merge
25μm
e
HAEC
VEC
VEL
Static
Flow
Cell numbers
0-20
20-40
40-60
60-80
80-90
Angle of alignment (degrees)

Supplementary Figure 6. Related to Figure 6. **a** Bright-field images of hPSC-derived VELs at passages 3/4/5. Note the fibroblastic morphology of VELs at passage 5. Scale bar: 25 µm. **b** Confocal images of hPSC-derived VELs at passages 3/4/5. Note that at passage 4, both CD31 and SMA were expressed, indicating of undergoing differentiation of hPSC-derived VELs. At passage 5, CD31 was markedly down-regulated, indicating of loss EC phenotype. Scale bar: 25 µm. **c** IF staining showing that hPSC-derived VELs express VE-cad and CD31. Scale bar: 25 µm. **d** IF staining of the newly identified markers for day 7 hPSC-derived VELs. Scale bar: 25 µm. **e** Quantification of cell orientation (positive value of degrees) for primary VECs, VELs and HAEC, under static and flow conditions (for panel d in Figure 6). Orientation of cells related to the flow direction. Thereby, 0 degree and 90 degree alignment angle represents parallel and perpendicular, respectively. At least 50 cells were evaluated per condition across 3 independent replicates by ImageJ. All experiments were repeated at least two times. Shown are representative data for panels a and b.

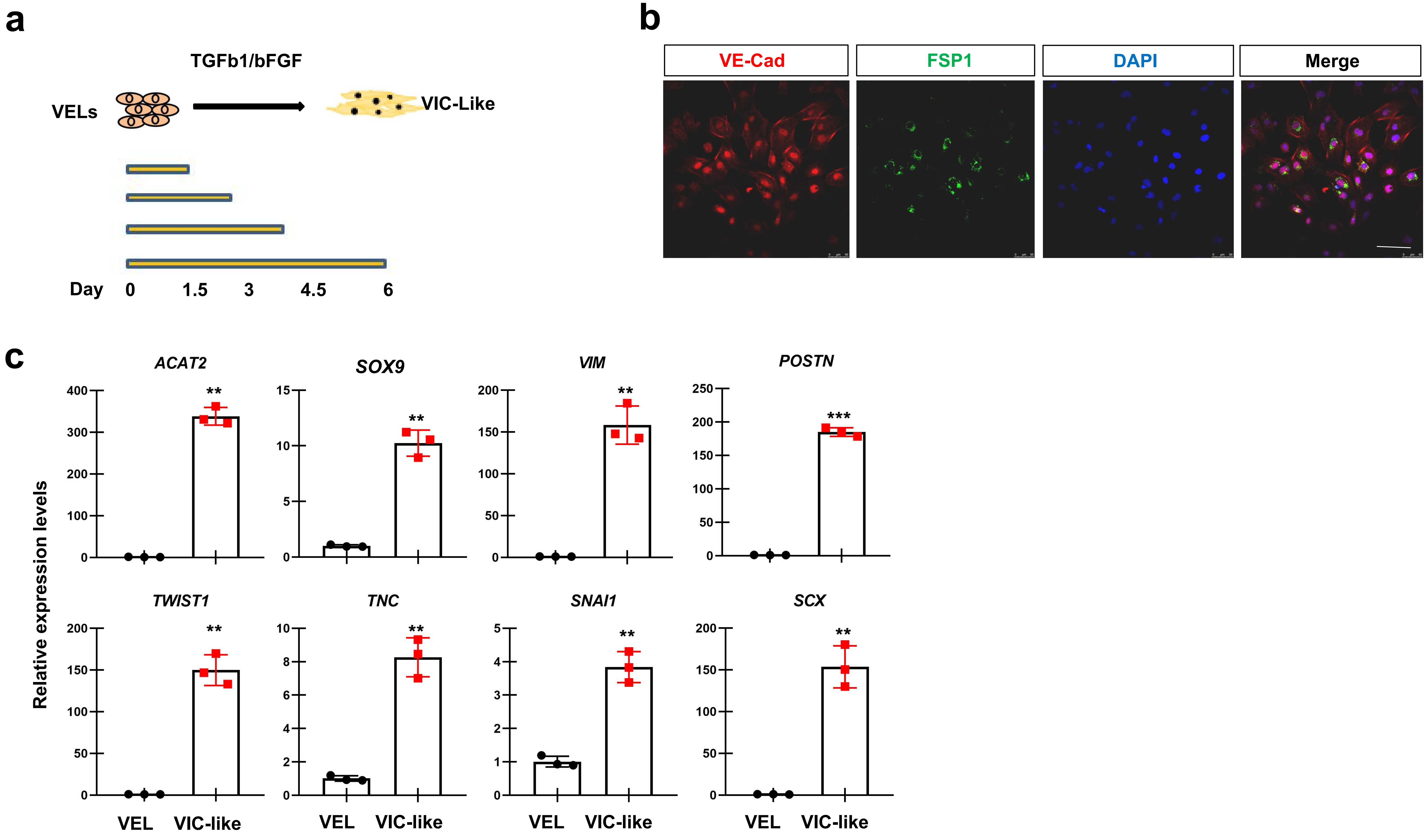

Supplementary Figure 7. Related to Figure 7. **a** A cartoon diagram showing the experimental design for converting hPSC-derived VELs to VIC-like cells. **b** IF staining of VE-cad and FSP1 for hPSC-derived VIC-like cells. Scale bar: 100 µm. **c** The qRT-PCR analysis of indicated VIC markers for hPSC-derived VELs that were under the combined treatment with high concentration TGFb1 and bFGF for 4.5 days. All experiments were repeated 3 times. The paired t test in Graphpad software was used for the statistical analysis. Significant levels are: *$p < 0.05$; **$P < 0.01$; ***$P < 0.001$. Shown are representative images for panel b.

a

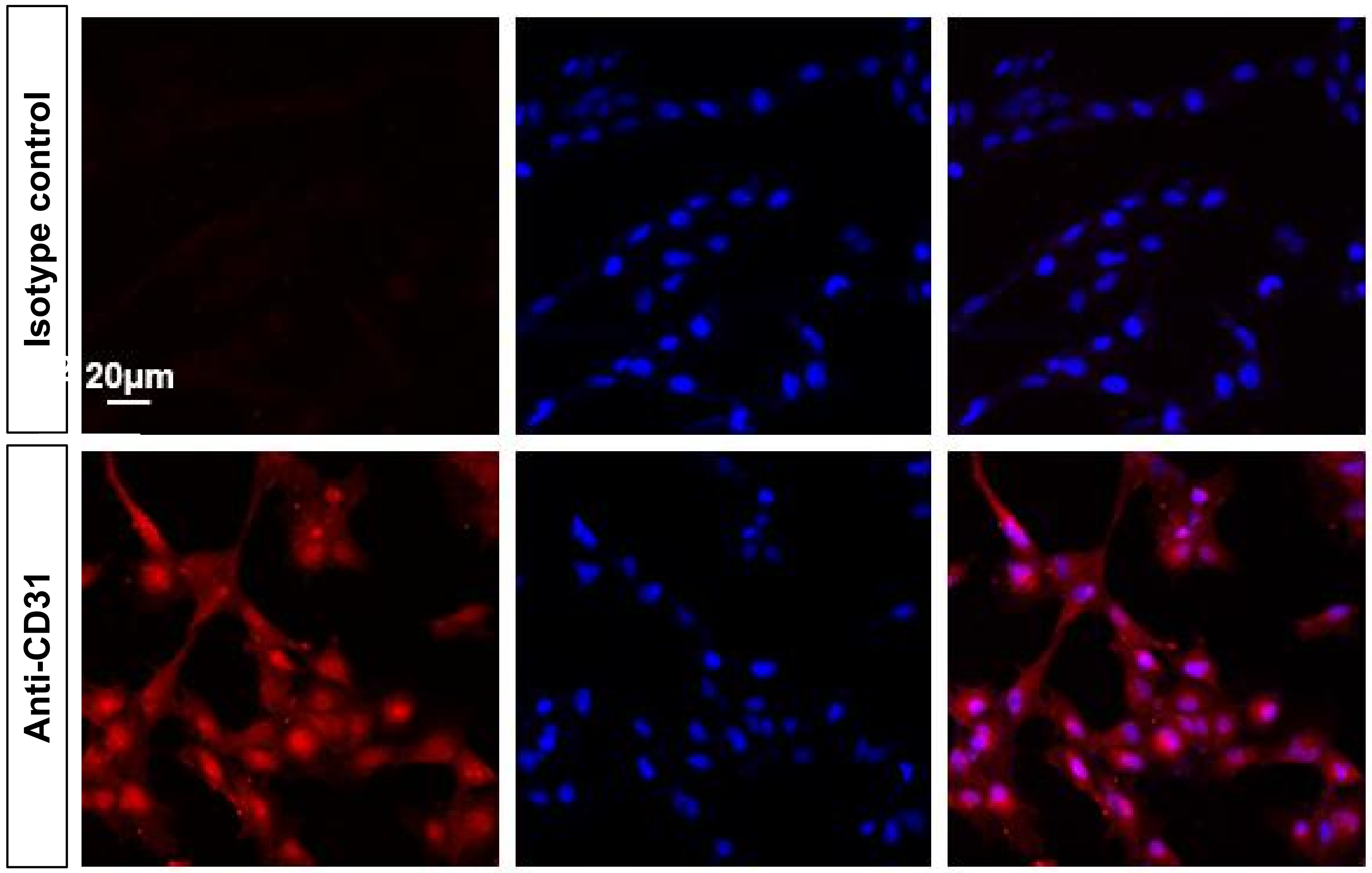

b

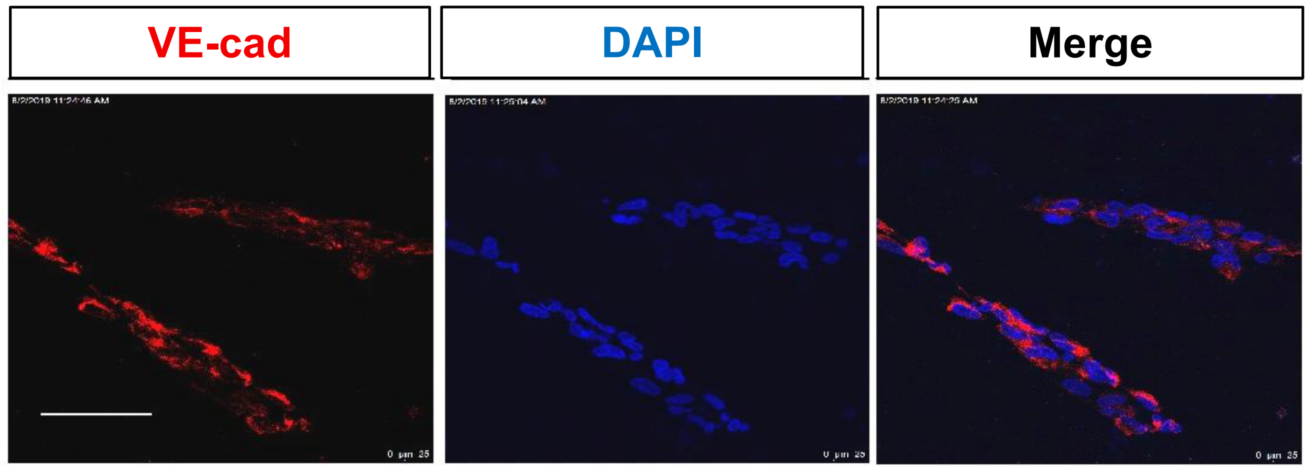

Supplementary Figure 8. Related to Figure 8. **a** IF staining of CD31 in hPSC-derived VELs seeded on the DCVs. Scale bar: 20 μm. **b** IF staining of VE-cad for hPSC-derived VELs after seeded on the DCVs. Scale bar: 50 μm. All experiments were repeated 3 times. Shown are representative images for panel a and b.
