# Supplementary material for: Generation and characterization of cardiac valve endothelial-like cells from human pluripotent stem cells
Source: Commun Biol. 2021 Sep 6;4:1039. doi: 10.1038/s42003-021-02571-7 (PMC8421482; doi:10.1038/s42003-021-02571-7)
Supplement: Supplementary file 3 — Description of Additional Supplementary Files [file 42003_2021_2571_MOESM3_ESM.pdf]

Description of Additional Supplementary Files

File name: Supplementary Data 1

Description: Detailed data for Figures 1b, 1d, 1e, Supplementary Figure 1c, Figures 2b, 2c, 2h, 3e, 4b, 4c, 4d, 4i, 5b, 5c, 6b, 6f, 6g, 7a, 7c, 7d, Supplementary Figure 7c, Figures 8b, 8c.
