# Supplementary material for: Generation and characterization of cardiac valve endothelial-like cells from human pluripotent stem cells
Source: Commun Biol. 2021 Sep 6;4:1039. doi: 10.1038/s42003-021-02571-7 (PMC8421482; doi:10.1038/s42003-021-02571-7)
Supplement: Supplementary file 5 — Reporting summary [file 42003_2021_2571_MOESM5_ESM.pdf]

nature research

Corresponding author(s): yuhua Sun

Last updated by author(s): Aug 9, 2021

Reporting Summary

Nature Research wishes to improve the reproducibility of the work that we publish. This form provides structure for consistency and transparency in reporting. For further information on Nature Research policies, see our Editorial Policies and the Editorial Policy Checklist.

Statistics

For all statistical analyses, confirm that the following items are present in the figure legend, table legend, main text, or Methods section.

n/a	Confirmed	
☐	☒	The exact sample size (n) for each experimental group/condition, given as a discrete number and unit of measurement
☐	☒	A statement on whether measurements were taken from distinct samples or whether the same sample was measured repeatedly
☐	☒	The statistical test(s) used AND whether they are one- or two-sided<br>*Only common tests should be described solely by name; describe more complex techniques in the Methods section.*
☒	☐	A description of all covariates tested
☐	☒	A description of any assumptions or corrections, such as tests of normality and adjustment for multiple comparisons
☐	☒	A full description of the statistical parameters including central tendency (e.g. means) or other basic estimates (e.g. regression coefficient) AND variation (e.g. standard deviation) or associated estimates of uncertainty (e.g. confidence intervals)
☐	☒	For null hypothesis testing, the test statistic (e.g. F, t, r) with confidence intervals, effect sizes, degrees of freedom and P value noted<br>*Give P values as exact values whenever suitable.*
☒	☐	For Bayesian analysis, information on the choice of priors and Markov chain Monte Carlo settings
☒	☐	For hierarchical and complex designs, identification of the appropriate level for tests and full reporting of outcomes
☒	☐	Estimates of effect sizes (e.g. Cohen's d, Pearson's r), indicating how they were calculated

Our web collection on statistics for biologists contains articles on many of the points above.

Software and code

Policy information about availability of computer code

Data collection	No software used to collect data. Conversion to fastq format of bulk RNA-seq was performed by BGI, Shenzhen China.
Data analysis	Data were analyzed using Student’s t-test or paired t-test analysis. Error bars represent s.e.m. RNA-seq and scRNA-seq data analysis were done by BGI, Shenzhen China. DEGs were defined by FDR < 0.05 and a Log2 fold change >1. Image J software (version 1.45) was used for analysis of WB and IF data. Analysis of FACs data was done by FlowJo 10.5.0 (TreeStar, OR, USA).

For manuscripts utilizing custom algorithms or software that are central to the research but not yet described in published literature, software must be made available to editors and reviewers. We strongly encourage code deposition in a community repository (e.g. GitHub). See the Nature Research guidelines for submitting code & software for further information.

Data

Policy information about availability of data

All manuscripts must include a data availability statement. This statement should provide the following information, where applicable:

- Accession codes, unique identifiers, or web links for publicly available datasets
- A list of figures that have associated raw data
- A description of any restrictions on data availability

All RNA-seq data are available at https://bigd.big.ac.cn/. The accession number is PRJCA002549. All other related data will be available upon reasonable request.

Field-specific reporting

Please select the one below that is the best fit for your research. If you are not sure, read the appropriate sections before making your selection.

☒ Life sciences ☐ Behavioural & social sciences ☐ Ecological, evolutionary & environmental sciences

For a reference copy of the document with all sections, see nature.com/documents/nr-reporting-summary-flat.pdf

Life sciences study design

All studies must disclose on these points even when the disclosure is negative.

Sample size	All experiments were conducted with at least two independent biological replicate cell lines and multiple biological replicates. For full list of cell lines and isolated valves from human hearts used in this study,please see supplementary information. Sample sizes were determined based on our previous experience and similar studies of other groups.
Data exclusions	No data exclusion was done.
Replication	All experiments were performed for al least two times. Q-PCR and IF were performed al least three times to satisfy significance analysis. Data presented are either representative of of replicates experiments of similar finding or average of replicates as indicated. Reported results were repeated and confirmed for at least two independent experiments. Key experimental findings were reproduced by two investigators involved in the lab.
Randomization	Samples were allocated into experimental groups according genotype or cell types.
Blinding	The investigators were blinded to group allocation during data production, but were not blinded to group allocation during data analysis, for stem cell work described in the manuscript.

Behavioural & social sciences study design

All studies must disclose on these points even when the disclosure is negative.

Study description	
Research sample	
Sampling strategy	
Data collection	
Timing	
Data exclusions	
Non-participation	
Randomization	

Ecological, evolutionary & environmental sciences study design

All studies must disclose on these points even when the disclosure is negative.

Study description	
Research sample	
Sampling strategy	
Data collection	
Timing and spatial scale	
Data exclusions	

Reproducibility

Randomization

Blinding

Did the study involve field work? ☐ Yes ☐ No

Field work, collection and transport

Field conditions

Location

Access & import/export

Disturbance

Reporting for specific materials, systems and methods

We require information from authors about some types of materials, experimental systems and methods used in many studies. Here, indicate whether each material, system or method listed is relevant to your study. If you are not sure if a list item applies to your research, read the appropriate section before selecting a response.

Materials & experimental systems

n/a	Involved in the study
☐	☒ Antibodies
☐	☒ Eukaryotic cell lines
☒	☐ Palaeontology and archaeology
☒	☐ Animals and other organisms
☒	☐ Human research participants
☒	☐ Clinical data
☒	☐ Dual use research of concern

Methods

n/a	Involved in the study
☒	☐ ChIP-seq
☐	☒ Flow cytometry
☒	☐ MRI-based neuroimaging

Antibodies

Antibodies used

Anti-NFATc1 (Abcam, ab2796); Anti-ISL1 (Abcam, ab178400); Anti-VE-cadherin (Abcam, ab33168), Anti-CD31 (R&D, FAB35679), Anti-KDR (R&D, FAB3579), Anti-CDH5 (BD Horizon, 561569), Anti-SOX9 (Proteintech, 67439-1-Ig), Anti-HEY1 (Proteintech, 19929-1-AP), Anti-JAG1 (Proteintech, 668909-1-Ig) or (Invitrogen, PA5-86057), Anti-NOTCH1 (Proteintech, 20687-1-AP), Anti-NOTCH4 (Abcam, ab225329), Anti-P-SELECTIN (Proteintech, 60322-1-Ig) and the isotype control antibodies: mouse IgG (R&D�C002P) and rabbit IgG (Invitrogen, 10500C). ISL1 (Abcam, ab178400), CD31 (Abcam, ab28364), NOTCH4 (Abcam, ab225329), DLL4 (Abcam, ab7280); VE-cadherin (Abcam, ab33168), CDH5 (proteintech, 66804-4-lg), NFATc1 (Abcam, ab2796), Endomucin (Abcam, ab106100), HEY1 (Proteintech, 19929-1-AP), LDB2 (Abcam, ab3627), GATA4 (Proteintech, 19530-1-AP) , KLF9 (Genetex, GTX129315) ,Anti-PROX1 (Proteintech, 11067-2-AP) and NESTIN (Abcam, ab22035).

Validation

IF and flow antibodies used are chosen based on the published work in the field. And each antibody was validated for Western blot - correct size of the detected bands based on the protein marker, as shown in the figures of this manuscript.

Eukaryotic cell lines

Policy information about cell lines

Cell line source(s)

Human endothelial cell line HUVEC (#CRL-1730) was purchased from the American Type Culture Collection (Rockville, MD, USA). Human aortic endothelial cell line HAEC (#6100) was purchased from Shanghai Cell Bank, CAS (Shanghai, China). PGP1 human induced pluripotent stem cells (kindly provided by Prof. Zhang from Hubei University, Wuhan, China), and human ESC lines (H8 and H9 lines, Wicell, WI, USA).

Authentication

Genotyping followed by Sanger sequencing, followed by qPCR or Western blot

Mycoplasma contamination

tested negative

Commonly misidentified lines
(See ICLAC register)

no

Palaeontology and Archaeology

Specimen provenance

Specimen deposition

Dating methods

☐ Tick this box to confirm that the raw and calibrated dates are available in the paper or in Supplementary Information.

Ethics oversight

Note that full information on the approval of the study protocol must also be provided in the manuscript.

Animals and other organisms

Policy information about studies involving animals; ARRIVE guidelines recommended for reporting animal research

Laboratory animals

Wild animals

Field-collected samples

Ethics oversight

Note that full information on the approval of the study protocol must also be provided in the manuscript.

Human research participants

Policy information about studies involving human research participants

Population characteristics

Recruitment

Ethics oversight

Note that full information on the approval of the study protocol must also be provided in the manuscript.

Clinical data

Policy information about clinical studies

All manuscripts should comply with the ICMJE guidelines for publication of clinical research and a completed CONSORT checklist must be included with all submissions.

Clinical trial registration

Study protocol

Data collection

Outcomes

Dual use research of concern

Policy information about dual use research of concern

Hazards

Could the accidental, deliberate or reckless misuse of agents or technologies generated in the work, or the application of information presented in the manuscript, pose a threat to:

No	Yes	
☐	☐	Public health
☐	☐	National security
☐	☐	Crops and/or livestock
☐	☐	Ecosystems
☐	☐	Any other significant area

Experiments of concern

Does the work involve any of these experiments of concern:

No	Yes	
☐	☐	Demonstrate how to render a vaccine ineffective
☐	☐	Confer resistance to therapeutically useful antibiotics or antiviral agents
☐	☐	Enhance the virulence of a pathogen or render a nonpathogen virulent
☐	☐	Increase transmissibility of a pathogen
☐	☐	Alter the host range of a pathogen
☐	☐	Enable evasion of diagnostic/detection modalities
☐	☐	Enable the weaponization of a biological agent or toxin
☐	☐	Any other potentially harmful combination of experiments and agents

ChIP-seq

Data deposition

☐ Confirm that both raw and final processed data have been deposited in a public database such as GEO.

☐ Confirm that you have deposited or provided access to graph files (e.g. BED files) for the called peaks.

Data access links<br>*May remain private before publication.*	
Files in database submission	
Genome browser session<br>(e.g. UCSC)	

Methodology

Replicates	
Sequencing depth	
Antibodies	
Peak calling parameters	
Data quality	
Software	

Flow Cytometry

Plots

Confirm that:

☒ The axis labels state the marker and fluorochrome used (e.g. CD4-FITC).

☒ The axis scales are clearly visible. Include numbers along axes only for bottom left plot of group (a 'group' is an analysis of identical markers).

☒ All plots are contour plots with outliers or pseudocolor plots.

☒ A numerical value for number of cells or percentage (with statistics) is provided.

Methodology

Sample preparation	Cells were washed twice with DPBS (BI, China), and digested with Typsin (BI) for 1 min, folowed by wash with DPBS containing 0.5% BSA (0.5% PBSA). Next, cells were centrifuged and washed once with 0.5% PBSA. The resuspended cells in 200 ul 0.5% PBSA were passed the flow tube to obtain single cells. After that, cells were incubated with the antibodies for 30 min at room temperature. After 3 times washing, cells were treated with secondary antibodies at room temperature for 15 min. Finally, cells were washed twice and resuspended with 300-500 μl 0.5% PBSA, and then analyzed by Accuri C6 flow cytometer (BD Biosciences, USA).
Instrument	BD AccuriC6 flow cytometer

Software	FlowJo 10.5.0 (TreeStar, OR, USA)
Cell population abundance	Abundance and purity in the relevant cell populations within post-sort fractions were assessed by flow cytometry analysis.
Gating strategy	The gating strategies used in the study will be added when it is required. Negative controls (isotype match antibodies or negative cells) were used to determines negative and positive boundaries.

☒ Tick this box to confirm that a figure exemplifying the gating strategy is provided in the Supplementary Information.

Magnetic resonance imaging

Experimental design

Design type	
Design specifications	
Behavioral performance measures	

Acquisition

Imaging type(s)	
Field strength	
Sequence & imaging parameters	
Area of acquisition	

Diffusion MRI ☐ Used ☐ Not used

Preprocessing

Preprocessing software	
Normalization	
Normalization template	
Noise and artifact removal	
Volume censoring	

Statistical modeling & inference

Model type and settings	
Effect(s) tested	

Specify type of analysis: ☐ Whole brain ☐ ROI-based ☐ Both

Statistic type for inference (See Eklund et al. 2016)	
Correction	

Models & analysis

n/a	Involved in the study
☐	☐ Functional and/or effective connectivity
☐	☐ Graph analysis
☐	☐ Multivariate modeling or predictive analysis

Functional and/or effective connectivity	
Graph analysis	
Multivariate modeling and predictive analysis	
